# Tongue and jaw movement assessed by 3D motion capture during gum chewing

**DOI:** 10.3389/fphys.2024.1409005

**Published:** 2024-08-28

**Authors:** Rikako Sato, Shohei Kodama, Jumpei Okawa, Kazuhiro Murakami, Takahiro Ono, Kazuhiro Hori

**Affiliations:** ^1^ Division of Comprehensive Prosthodontics, Faculty of Dentistry and Graduate School of Medical and Dental Sciences, Niigata University, Niigata, Japan; ^2^ Department of Dysphagia Rehabilitation, Asahi University School of Dentistry, Gifu, Japan; ^3^ Department of Gerodontology, Osaka Dental College, Osaka, Japan

**Keywords:** tongue movement, jaw movement, three-dimension, chewing, chewing cycle, motion capture

## Abstract

**Introduction:**

The tongue plays an important role in mastication, swallowing, and articulation, but it cannot be directly observed because of its location inside the oral cavity. This study aimed to clarify detailed 3D tongue movements during chewing using electromagnetic articulography (EMA).

**Materials and Methods:**

The participants were 10 healthy, young volunteers (average age 26.8 ± 2.1 years; 5 males, 5 females). Tongue and jaw movement during gum chewing was measured and recorded using EMA. Four EMA sensors were attached to the anterior, posterior, left, and right surfaces of the tongue, and one sensor was also attached to the mandibular left incisor. The tongue motion during the chewing cycle was spatially and sequentially analyzed based on the motion trajectories of the tongue and mandible.

**Results and Discussion:**

The tongue moved downward and to the masticatory side in a manner similar to the movement of the jaw. The anterior tongue marker moved downward to a greater extent than the other tongue markers. However, the tongue moved forward as the jaw moved backward. The anterior marker reached the most anterior position during the jaw-opening phase and the posterior markers reached the most anterior position during the jaw-closing phase. Just before maximum jaw-opening, all markers on the tongue reached the bottom lowest position. During the jaw-closing phase, the tongue reached the dominant farthest position in the masticatory side. All the markers reached the most posterior position during the occlusal phase.

**Conclusion:**

These findings demonstrate the sequence of tongue motion patterns during gum chewing.

## 1 Introduction

The tongue plays an important role during mastication, swallowing, and articulation. In particular, the tongue makes complex movements in coordination with mandibular movements during mastication (Hori et al., 2006). The tongue repositions food from the oral cavity onto the dentition, helps to form the bolus, and transports the bolus to the pharynx (Taniguchi et al., 2013). When the tongue is damaged by aging, neuromuscular disease, or glossectomy, these movements are impaired (Wang et al., 2018; Depeyre et al., 2020). Therefore, tongue movements should be examined during swallowing and mastication to demonstrate the mechanism of dysphagia.

Because the lips are closed during function, the movement of tongue located inside the oral cavity cannot be observed directly. In the 1950s, Abd-el-Malek observed tongue movements during mastication in participants who had lost some of their teeth by parting the lips with small retractor forceps (Abd-El-Malek, 1955). Many years after this direct observation under unique circumstances, indirect image analysis of tongue movement during mastication was performed using ultrasound (US) (Imai et al., 1995; Remijn et al., 2015) or videofluorography (VF) (Taniguchi et al., 2013; Palmer et al., 1997; Mioche et al., 2002; Iida et al., 2023). Imai et al. observed tongue movement during mastication using US images, and reported that the tongue turned the food, mixed it with saliva, sorted out unsuitable particles, and aided in bolus formation (Imai et al., 1995). Palmer et al. reported that the amplitude and timing of tongue movements differ depending on the type of food, and recorded details of two-dimensional tongue movements using VF (Palmer et al., 1997). VF allows sequences from mastication to swallowing to be recorded, including the position of the bolus. However, disadvantages of this technique include the risk of radiation exposure and the fact that the two-dimensional images overlap organs other than the tongue, making it impossible to clearly observe the tongue.

Electromagnetic articulography (EMA) can capture the movement of the tongue surface at multiple points. It has a high sampling frequency and can quantitatively measure tongue movement with high precision (Lezcano et al., 2020). Additionally, EMA entails no exposure to radiation and is not as affected by the skills of the operator as US. We constructed a comprehensive tongue motor function evaluation system that simultaneously measures tongue movement and tongue pressure to observe tongue movements before and after tongue-palate contact. In our previous studies, we analyzed and reported the relationship between tongue movement and tongue pressure during water swallowing (Shitara et al., 2020; Kodama et al., 2021). Although efforts have been made to observe tongue movement during swallowing, few studies have analyzed tongue movement during chewing in detail in three dimensions.

We hypothesized that the tongue might coordinate with the movement of the jaw during chewing, enabling us to analyze the details of tongue movements during chewing using EMA. The purpose of this study was to clarify the details of three-dimensional tongue and jaw movements during gum chewing.

## 2 Materials and methods

### 2.1 Participants

This study was designed as an observational study. The participants were 10 healthy volunteers (5 men, 5 women, average age 26.8 ± 2.1 years). The participants were recruited from Niigata University staff and students who understood the purpose of this study and provided written consent. Exclusion criteria were those with missing teeth, toothache, a subjective or objective masticatory/swallowing disfunction, a history of temporomandibular joint disease, those undergoing orthodontic treatment, and those with latex allergies. This experimental protocol was approved by Niigata University’s Ethics Review Committee (2015–3,050).

### 2.2 Equipment

**FIGURE 1:**
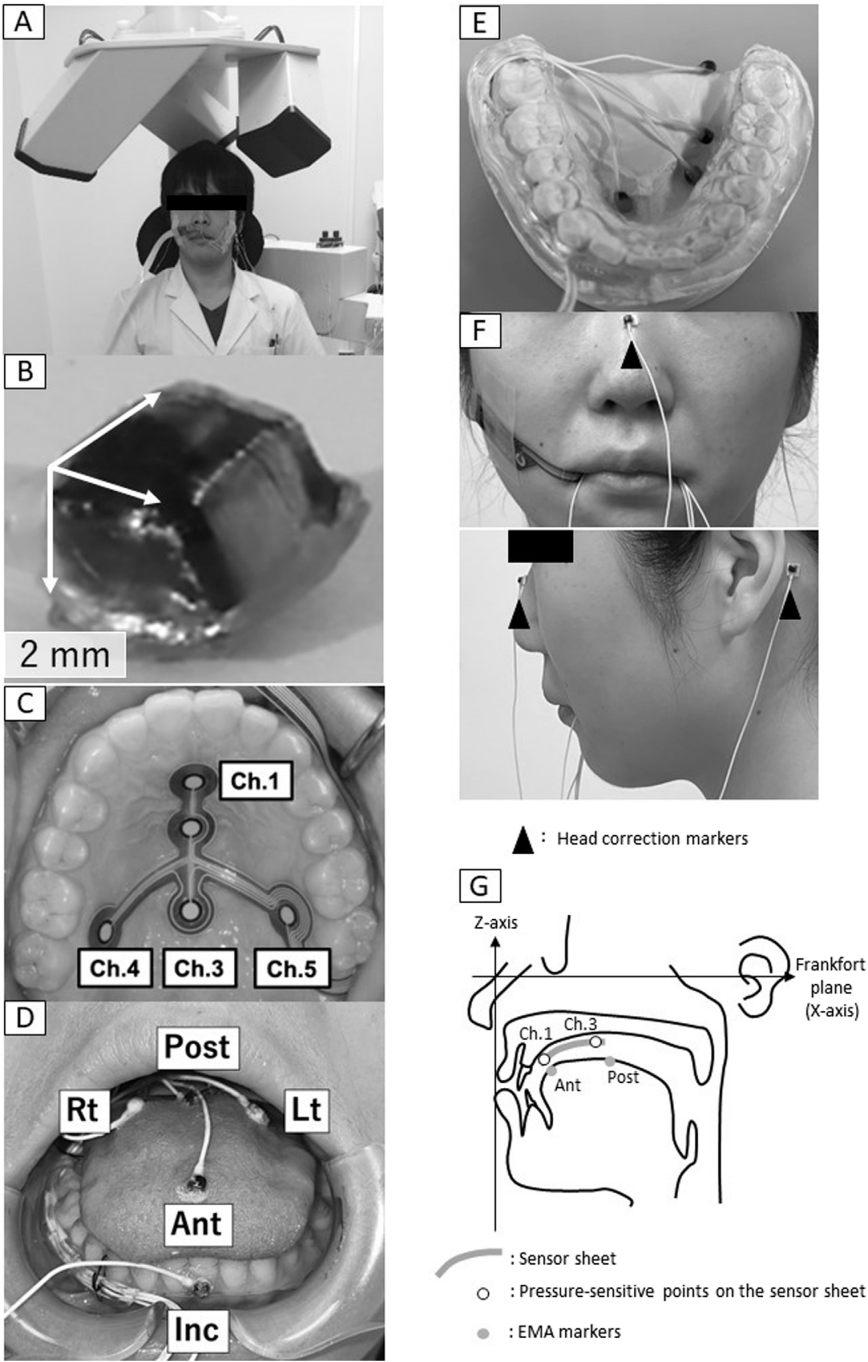
Electromagnetic articulography. **(A)** Measurement setup, **(B)** marker, **(C)** Sensor sheet, **(D)** Marker positions, **(E)** Experimental intraoral appliance, **(F)** Head correction markers, **(G)** Settings for construction of three-dimensional coordinates, Ant, anterior; Post, posterior; Lt, left; Rt, right; Inc, incisal.

EMA (AG501, Carstens, Göttingen, Germany, Figure 1A) was used to record the motion trajectory of the tongue and mandible. EMA can capture tongue and jaw motion in three dimensions. It is composed of three transmitters that create a magnetic field where measurements take place and cube-shaped makers (2 mm × 2 mm × 2 mm, Figure 1B) that provide position data. The marker is internally equipped with two coils with different numbers of turns, and a different value of induced current arises in each coil as the marker moves within the magnetic field. The strength of this induced current is dependent on the distance between the transmitter and the marker, and the potential difference between the two coils is converted to position data, resulting in a three-dimensional and real-time recording of the motion trajectory of the marker as it moves in the magnetic field. Prior to measurement, the EMA markers were fixed in a specified direction to the device for calibration, and they were moved in a specific manner within the magnetic field, thereby calibrating the position data and movement direction of each marker.

The EMA markers were affixed directly to the four points on the tongue, one point on the left incisal teeth for mandibular movement, and three points on the upper face for head correction using surgical adhesive (Aronalpha A, Toagosei Co., Ltd., Toyama, Japan). The four markers were positioned on the tongue at the anterior-median (Ant), posterior-median (Post), Posterior-left (Lt) and posterior-right (Rt) (Figure 1D). These four markers corresponded to the positions on a T-shaped tactile sensor sheet (Hori et al., 2009) attached to the palate (Figure 1C). The sensor sheet has with the following five pressure-sensitive points: the anterior midline of the hard palate (channel [Ch.] 1), the center midline of the hard palate (Ch. 2), the posterior midline of the hard palate (Ch. 3), the right posterior of the hard palate (Ch. 4) and the left posterior of the hard palate (Ch. 5) (Figure 1C). The position of the EMA markers was determined by referring to the position of the pressure points on the tongue pressure sensor sheet. That is, Ant: Ch.1, Post: Ch. 3, Rt: Ch. 4, Lt: Ch. 5. The EMA makers were attached with reference to. The sensor sheet with the most appropriate dimensions was selected from three sizes based on the size of the individual’s hard palate.

To avoid the cables from the markers being chewed during measurement, an experimental intraoral appliance (Figures 1D, E 0.9 mm thickness) was fabricated and placed in the lower jaw. First, the participants were taken upper and lower oral impressions by alginate impression and an experimental model was made of anhydrite. Next, after securing a gap for the EMA marker wire (0.8 mm diameter) to passing from the right premolar to the distal molar, this appliance was made by a plastic disk (1.0 mm, erkodule, Erkodent) using a suction shaping machine (Erkoform, Erkodent). The occlusal surface of the appliance was ground down for being able to chew and an intraoral appliance was fabricated to cover the buccal and lingual sides of the mandibular dentition.

An additional marker (Inc.) was affixed to the labial surface of the lower left incisors. As reference points to correct for head movement, three markers were affixed, one to the middle of the nasal dorsum along the Frankfort plane and one each on the skin surface of the left and right mastoid process (Figure 1F).

In addition to the main measurement, we investigated whether these devices affect jaw movement (Supplemental experiment).

### 2.3 Measurement procedure

During measurement, the participants were seated in a chair with the head supported with a headrest so that the Frankfort plane was parallel to the floor. Both feet were placed flat on the floor. The sample food was a piece of tasteless and odorless gum (Saliva gum α, Tokyo Shizaisha Co. Ltd., Tokyo, Japan). The gum was softened in advance, and the participant was instructed to hold the gum on the tongue. A cue was given, and 50 chews were recorded. The participant was instructed to chew only on the left side, where the cable was not connected. The data obtained from the measurements were recorded on a PC at 250 Hz.

A preliminary experiment was conducted with the same settings 1 week before the experiment day for the purpose of adaptation.

### 2.4 Analysis

The chewing cycles that showed stable jaw movement were analyzed. The stable jaw movement cycle was defined as Class I and Class III according to Shiga et al.’s classification (Shiga et al., 2023), and as the cycle in which the jaw were clearly moving toward the masticatory side. A total of 290 cycles were analyzed. First, head motion was corrected by referring to the position of the head correction markers and three-dimensional coordinates were constructed. In the constructed three-dimensional coordinates (Figure 1G), the origin was set at the position of the head correction marker on the nose. The Frankfort plane was set as the X-axis (front–back direction; back is positive, front is negative) (Kodama et al., 2021). The line passing through the origin perpendicular to the X-axis and parallel to the left and right mastoid processes was defined as the Y-axis (positive on the right, negative on the left). The line perpendicular to the *X* and *Y*-axes was defined as the Z-axis (vertical direction; up is positive, down is negative).

**FIGURE 2:**
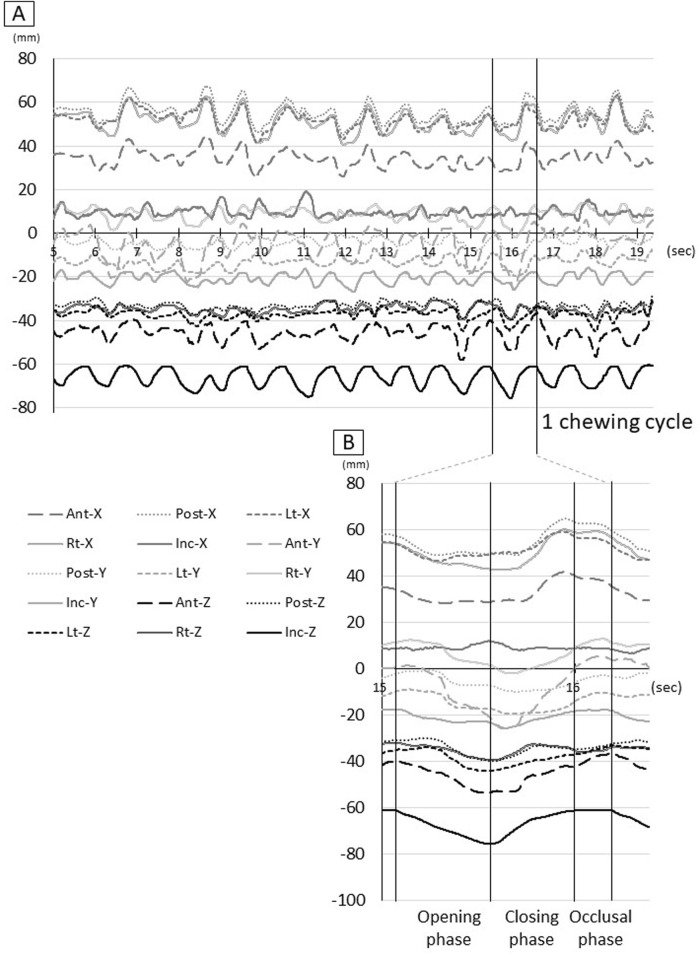
Raw waveform of tongue and jaw movement. **(A)** Waveform for all procedures, **(B)** Waveform for one chewing cycle, Ant: anterior, Post: posterior, Lt: left, Rt: right, Inc: incisal, X: *x*-axis, Y: *y*-axis, Z: *z*-axis.

A movie (Supplementary Movie; Supplementary Figure S1) of the three-dimensional coordinates and a time-series waveform graph (Figure 2A) of the tongue and incisor point markers during gum chewing were created. Next, referring to the movement of the Inc. marker, the period from the start of mouth opening to the end of one chewing cycle was set as 100% (Figure 2B), and all chewing movements were superimposed for each participant. Furthermore, the waveforms of all participants were superimposed. The movement trajectory of each marker was then drawn in frontal section (YZ plane) and sagittal section (XZ plane), and the movement was observed.

Next, during each chewing cycle, the maximum and minimum values of each marker on the *X*, *Y*, and Z-axes were calculated, and the movement range was calculated as the maximum value minus the minimum value. The times showing the maximum and minimum values were identified between normalized times (0%–100%).

### 2.5 Statistics

The maximum and minimum values and movement ranges during one chewing cycle on the *X*, *Y*, and Z-axes among the five markers (4 points on the tongue and 1 on the incisors) were compared using the Kruskal-Wallis test and the *post hoc* test with Bonferroni’s correction. The absolute maximum and minimum values of each marker were compared using the Mann-Whitney test.

The significance level was set at α = 0.05. SPSS software (version 28.0, IBM Japan, Tokyo, Japan) was used for all statistical analyses.

## 3 Results

### 3.1 Qualitative observation of trajectory

**FIGURE 3:**
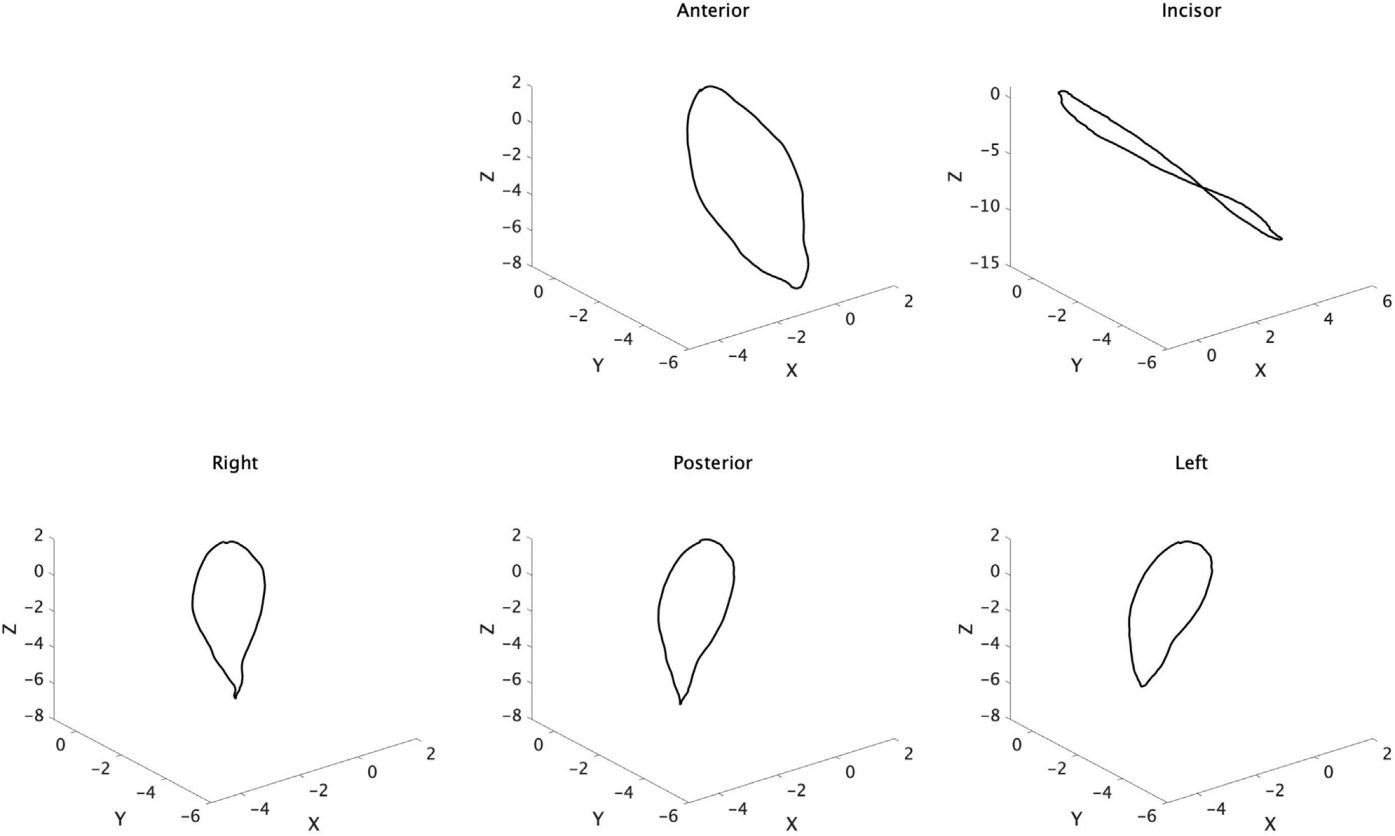
Trajectory of tongue and jaw movement in 3D view. Waveforms of all participants were normalized and synthesized.

Figure 3 shows the trajectory of each maker after normalized and synthesized processing. Three-dimensional analysis directly was complex, so we used the data of the reference plane for analysis.

#### 3.1.1 The trajectory of the frontal section

**FIGURE 4:**
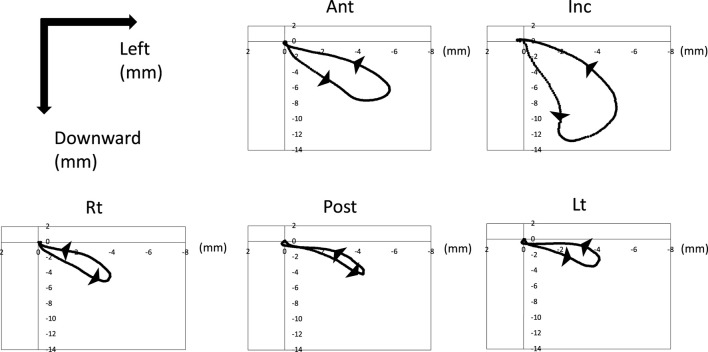
Trajectory of tongue and jaw movement in the frontal section. Waveforms of all participants were normalized and synthesized.

Figure 4 shows the trajectory of each marker on the YZ plane after normalized and synthesized processing (YZ plane, Figure 4). The mandible (Inc.) first moved downward, then moved to the left (the masticatory side) after reaching its maximum opening, and then returned to the central occlusal position. The marker on the tongue also moved downward, to the left, and then returned to its original position, similar to Inc. Ant at the front of the tongue had more dynamic movements than Lt, Post, and Rt at the posterior tongue.

#### 3.1.2 The trajectory of the sagittal section

**FIGURE 5:**
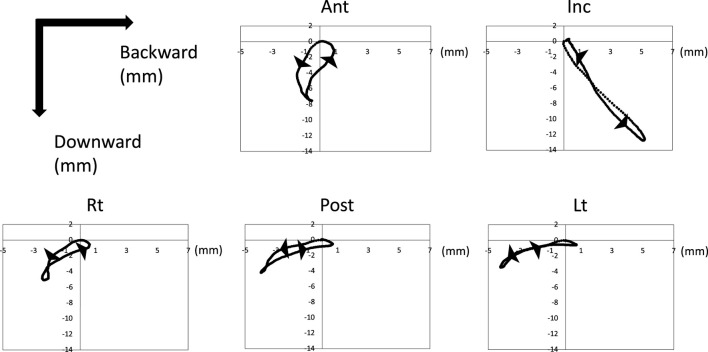
Trajectory of tongue and jaw movement in the sagittal section. Waveforms of all participants were normalized and synthesized.

Figure 5 shows the trajectory of each marker on the XZ plane after normalized and synthesized processing (XZ plane, Figure 5). The incisor point, Inc., hardly moved forward and moved only posteroinferiorly. However, the tongue mainly moved forward. In particular, Lt on the masticatory side moved mainly back and forth, with little downward movement. The trajectory of the horizontal section of each marker on the XY plane after normalized and synthesized processing was showed in Supplementary Figure S2.

### 3.2 The time course of tongue movement

**FIGURE 6:**
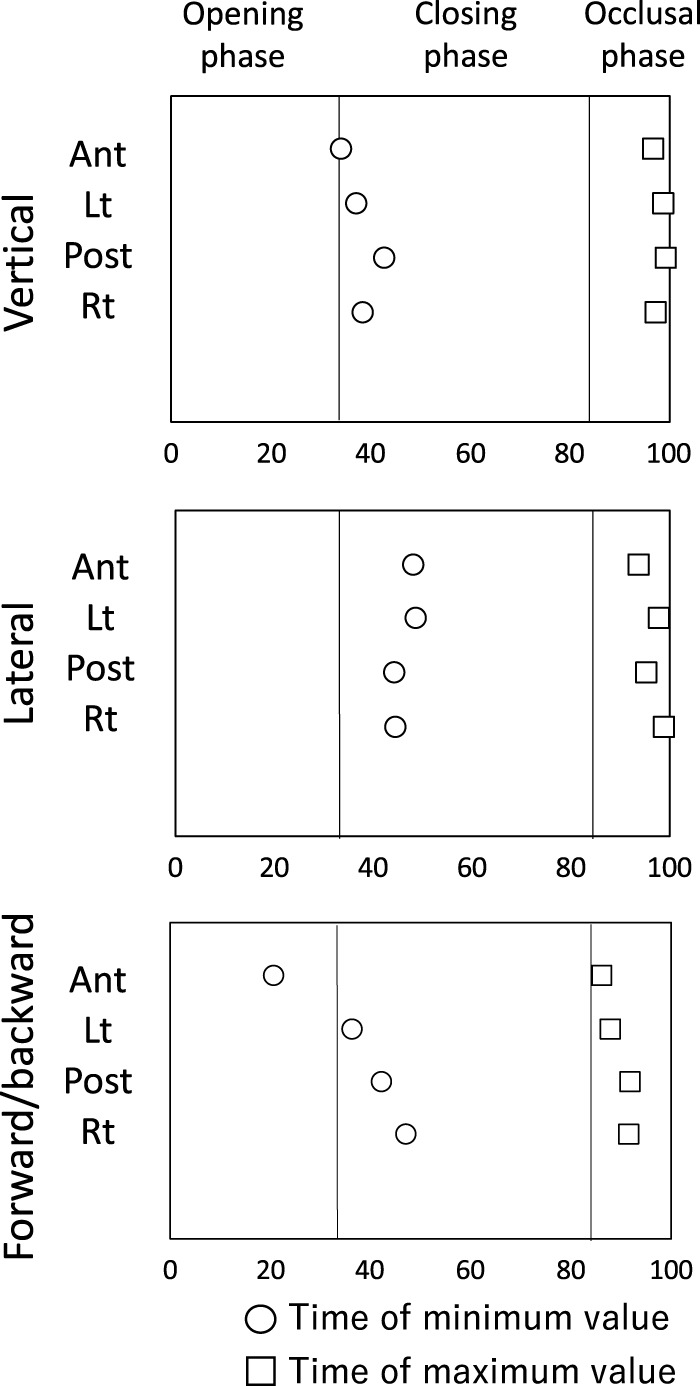
Schematic diagram of the time course of tongue movement during the chewing cycle. Time 0 was set as the start of the opening phase and 100 was set as the end of the occlusal phase. ○: time of minimum value, □: Time of maximum value.

Figure 6 shows the schematic diagram of the time course of tongue movement during the chewing cycle (Figure 6). Time 0 was set as the start of the opening phase and 100 was set as the end of the occlusal phase. The time at which the tongue movement reached its extreme value is also shown as a value from 0 to 100. The start of the closing phase (maximum mouth opening) was 32.9 ± 4.4 and the start of the occlusal phase was 83.7 ± 3.2.

#### 3.2.1 Vertical movement

The tongue moved downward as the mouth opened, reaching the lowest position near maximum mouth opening (Ant: 34.0 ± 7.0). Lt (37.3 ± 6.0), Post (42.8 ± 16.8), and Rt (38.3 ± 6.9) at the posterior tongue reached the lowest position slightly later than the time of maximum opening. After that, the tongue moved upward and assumed the uppermost position near the beginning of mouth opening.

#### 3.2.2 Lateral movement

As the mouth opened, the tongue moved to the left side (the masticatory side) and reached the leftmost position during the mouth closing phase (Ant: 47.8 ± 8.8, Lt: 48.4 ± 15.8, Post: 44.0 ± 19.0, Rt: 44.3 ± 16.3). The tongue then moved to the right and reached the rightmost position in the occlusal phase. All tongue markers were at the rightmost position just before mouth opening began (Ant: 93.7 ± 6.7, Lt: 97.6 ± 16.2, Post: 95.0 ± 12.4, 98.6 ± 8.7).

#### 3.2.3 Forward and backward movement

Ant reached the frontmost position before maximum mouth opening (Ant: 20.5 ± 18.3), and then Ant started moving backwards earlier than the marker on the posterior tongue. The posterior tongue markers (Lt, Post, Rt) reached the most anterior position during the closing phase (Lt: 36.2 ± 12.6, Post: 42.0 ± 24.7, Rt: 47.0 ± 31.3), and then moved backward during closing phase. All tongue markers reached the most posterior position during the occlusal phase (Ant: 86.1 ± 27.4, Lt: 87.7 ± 8.1, Post: 91.6 ± 9.5, Rt: 91.4 ± 26.2).

### 3.3 The amount and range of tongue movement

#### 3.3.1 Vertical movement

**FIGURE 7:**
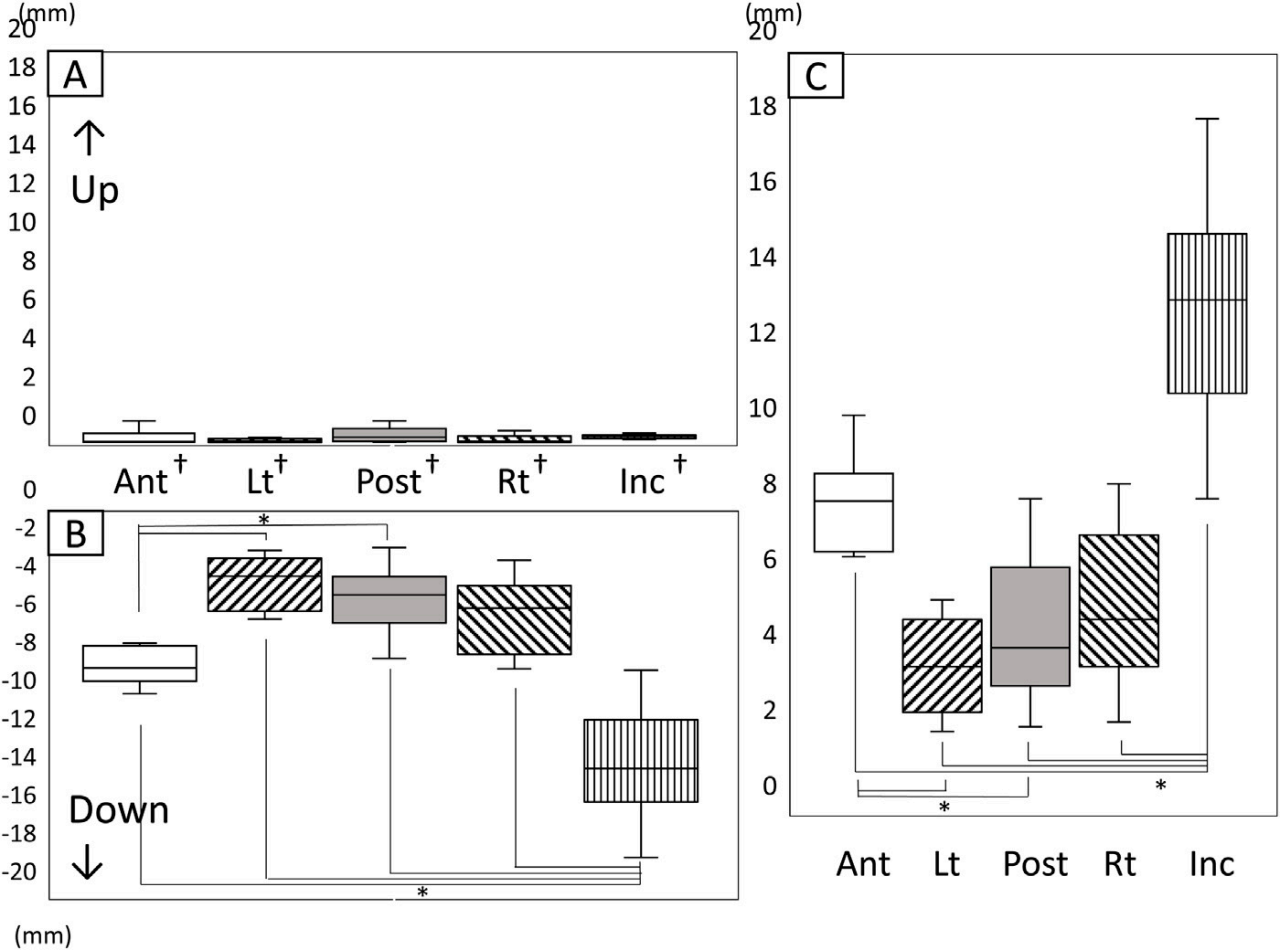
Amount and range of upward and downward tongue and jaw movement during the chewing cycle. **(A)** Amount of upward movement, **(B)** Amount of downward movement, **(C)** Range of vertical movement, *: significant difference among markers (*P* < 0.05), †: significant difference between upward and downward movement (*P* < 0.05), n.s: not significant, Ant: anterior, Post: posterior, Lt: left, Rt: right, Inc: incisal.

Figure 7 shows the amount and range of upward and downward tongue and jaw movement during the chewing cycle (Figure 7). The tongue markers hardly moved upward (Figure 7A), and the amount of downward movement was significantly greater than the upward movement (Figures 7A, B *P* < 0.05). The amount of downward movement of Inc. was also significantly greater than that of the tongue markers (P < 0.05).

The vertical movement range of Inc. was significantly greater than that of all tongue markers (Figure 7C). Additionally, the amount of downward movement of Lt on the masticatory side and Post at the posterior median of the tongue was significantly smaller than that of Ant at the front of the tongue.

#### 3.3.2 Lateral movement

**FIGURE 8:**
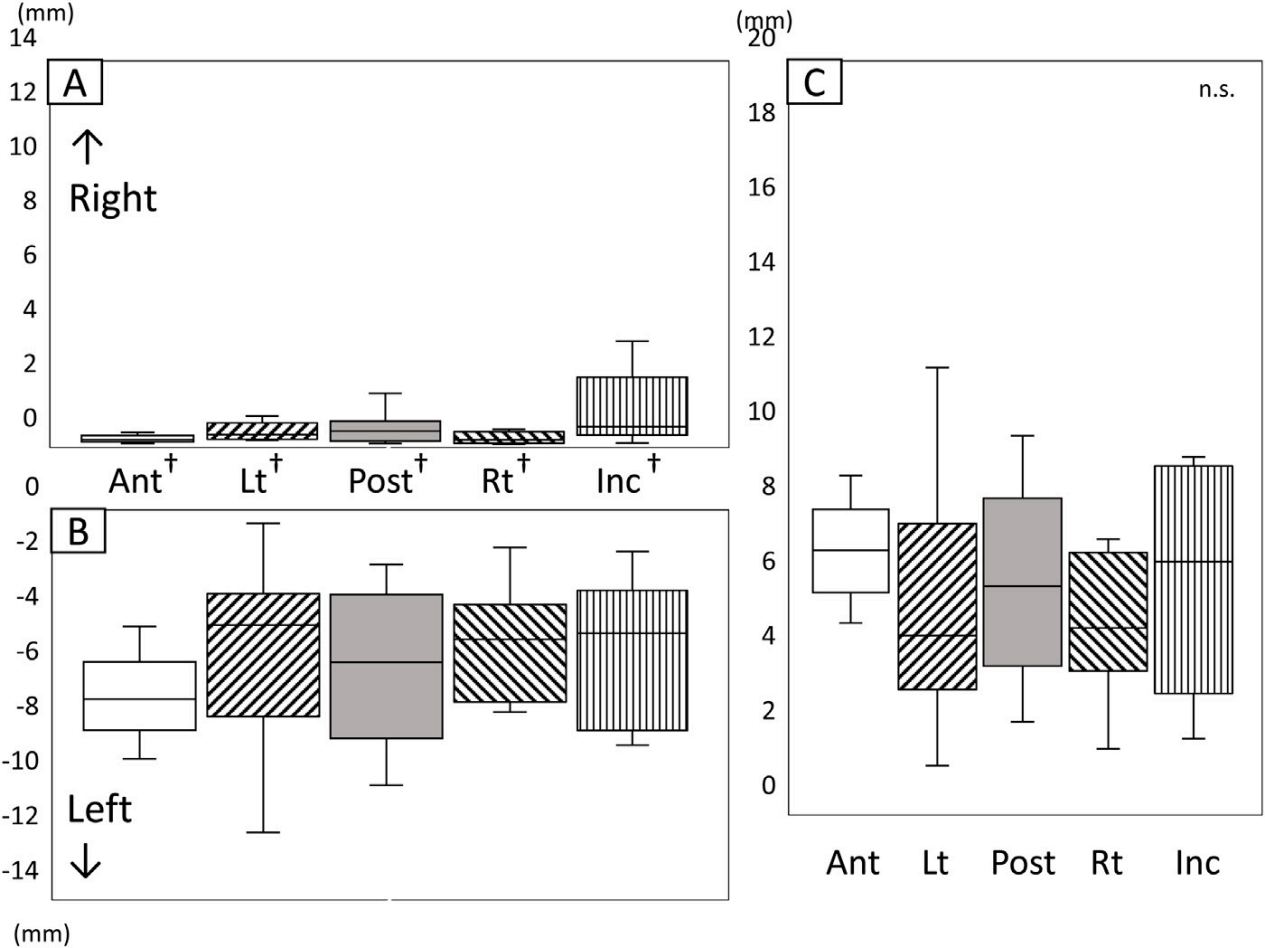
Amount and range of lateral tongue and jaw movement during the chewing cycle. **(A)** Amount of movement to the right, **(B)** Amount of movement to the left, **(C)** Range of lateral movement, *: Significant difference among markers (*P* < 0.05), †: significant difference between movement to right and left (*P* < 0.05), n.s: not significant, Ant: anterior, Post: posterior, Lt: left, Rt: right, Inc: incisal.

Figure 8 shows the amount and range of lateral tongue and jaw movement during the chewing cycle (Figure 8). The amount of lateral movement of both the lower jaw and the tongue was greater to the left (the masticatory side) than to the right (Figures 8A, B, P < 0.05). No significant differences were observed among any of the markers regarding the amount of movement toward the masticatory side (left side), the amount of movement toward the equilibrium side (right side), and the amount of range in the lateral direction (Figure 8C).

#### 3.3.3 Forward and backward movement

**FIGURE 9:**
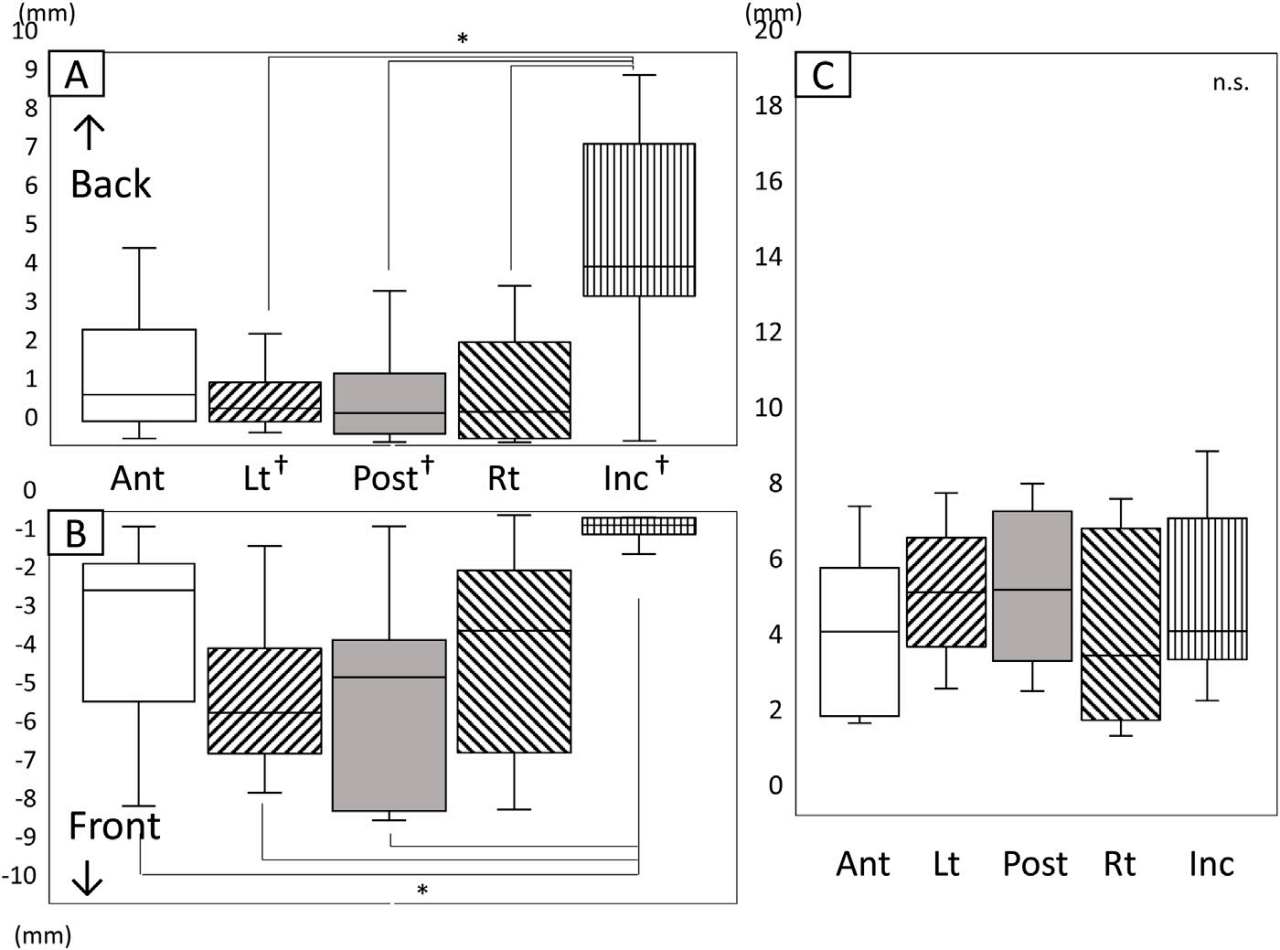
Amount and range of forward and backward tongue and jaw movement during the chewing cycle. **(A)** Amount of backward movement, **(B)** Amount of forward movement, **(C)** Range of forward/backward movement, *: Significant difference among markers (*P* < 0.05), †: Significant difference between forward and backward movement (*P* <0.05), n.s: not significant, Ant: anterior, Post: posterior, Lt: left, Rt: right, Inc: incisal.

Figure 9 shows the amount and range of forward and backward tongue and jaw movement during the chewing cycle (Figure 9). Inc. moved mainly backwards, whereas the tongue moved more forward than backward. The amount of backward movement of Inc. was significantly greater than the forward movement (Figures 9A, B, *P* < 0.05). The amount of forward movement of Lt and Post was significantly greater than the backward movement (P < 0.05).

Inc. recorded a significantly greater amount of backward movement than Lt, Post, and Rt (Figure 9A, P < 0.05). The amount of forward movement for Ant, Lt, and Post was greater than that of Inc. (Figure 9B). No significant differences were observed among all markers in the range of movement in an anteroposterior direction (Figure 9C).

## 4 Discussion

In this study, we measured tongue movement during gum chewing using EMA. To our knowledge, few studies have measured the movement of multiple points on the tongue in three dimensions with a high measurement frequency. Detailed analysis of tongue movements during function would be useful for elucidating the mechanism of mastication and swallowing, and the pathology of disorders.

Because the tongue exists within the oral cavity and the lips are closed during function, it is generally difficult to observe tongue movements directly during mastication and swallowing. To date, image analyzers such as VF (Palmer et al., 1997; Mioche et al., 2002) and US (Imai et al., 1995; Remijn et al., 2015) have been used to examine tongue movement during mastication. These devices can monitor the movement not only of the tongue but also the perioral organs. Additionally, the position of the bolus can be determined, although a contrast agent may be required. However, analysis may be difficult if the images overlap with other organs. Because it is difficult to quantitatively analyze the movement of fixed points from video footage, methods using markers are preferable. Furthermore, VF and US are limited to two-dimensional analysis. Another disadvantage of VF is the risk of radiation exposure. Although there is no risk of radiation exposure with US, it is necessary to devise ways to hold the probe in an appropriate position, operator skills are required, and the images have low temporal resolution.

The EMA we used in this study is a device that can make frequent quantitative measurements of the three-dimensional positions of multiple points in the oral cavity. Because each marker has a conductor, it has been mainly used to analyze tongue movement during pronunciation (Kim et al., 2014) and swallowing (Shitara et al., 2020; Kodama et al., 2021; Steele and Van Lieshout, 2008). In this study, by fabricating an appliance in the mandible, we succeeded in measuring tongue movement during gum chewing without interfering with the occlusion. Although it is possible that the appliance and conductor slightly inhibited natural chewing movements, the novelty and benefits of this study were that we were able to quantitatively analyze tongue and mandibular movements during chewing over time.

Few studies have recorded tongue movements during mastication. Abd-el-Malek (Abd-El-Malek, 1955) gathered participants who had lost some of their teeth and observed tongue movement during mastication by parting the lips with small retractor forceps. The tongue twisted over on one side, turning through a right angle so that its dorsum faced the lingual surface of the teeth. In this way, direct observation was carried out, but in very limited circumstances with only qualitative observation.

US allows the amount of tongue movement to be measured. Imai et al. reported that the tongue moved around 7–8 mm in the vertical dimension during mastication of a peanut (Imai et al., 1995). While only one position of the tongue was analyzed, these results are not significantly different from our results. However, it is known that jaw movement changes depending on the size, shape, and properties of food (Bhatka et al., 2004). It is possible that tongue movements differ when the food changes, and this needs to be investigated in future research.

In our previous study by Hori et al. (2006), tongue pressure measured during mastication reached its maximum value near the beginning of mouth opening. The results of the present study also revealed that the tongue was positioned at its uppermost position near the beginning of mouth opening.

Despite the risk of radiation exposure, VF is still considered to be a useful method for observing biological movements during mastication and swallowing. Markers are required to analyze tongue movements in detail in using VF. It is possible to observe the movement not only of the tongue, but also of the hyoid bone (Matsuo and Palmer, 2010) and soft palate (Matsuo et al., 2005), enabling the coordination with related organs to be studied. Mioche et al. observed tongue movement with VF and reported that the tongue pushed and rotated the bolus, and then placed it on the surface of the mandibular post-canine teeth during mastication (Mioche et al., 2002). They named this phenomenon “tongue-pushing (TP)” (Mioche et al., 2002). Iida et al. (2023) noted differences in tongue movement between TP and non-TP movement and that the tongue pushed the bolus on to the occlusal surface during the opening phase. This TP movement was the same as the jaw movement in the lateral dimension, indicating that both tongue and jaw movement caused the bolus to be moved to the masticatory side. Our results suggest that the tongue moves to the masticatory side from the opening phase and reaches the leftmost side during closing phase. The bolus seems to remain in place on the dentition because these tongue movements act like a wall. The cycles we analyzed in this study could be considered tongue-pushing cycles. We ultimately targeted 58% (290/500) cycles for analysis, which is consistent with the TP percentage (58.8%) reported by Iida et al. (2023). In future research, we aim to analyze non-TP cycles and explore the differences between TP and non-TP cycles.

Palmer et al. (1997) used VF to observe tongue movement in two dimensions during mastication and reported that the tongue moves forward and downward as the mouth opens, moves to the frontmost position during the opening movement, and moves up and back to the rearmost position as the mouth closes. In this study we recorded the movement of the tongue in three dimensions simultaneously. Because the tongue is located within the mandible, tongue movement is closely related to jaw movement. During the opening phase, the tongue moved forward, downward, and to the left (masticatory side). Additionally, the tongue in the horizontal dimension moved to the front; however, the jaw moved backward. This movement might reflect the tongue scooping up the gum that was crushed by the dentition during the previous occlusal phase. At maximum opening, the tongue reached its lowest position. At this time, Lt was located at a higher position than the other posterior markers (although the difference was not significant), suggesting that the tongue functions like a wall to prevent the gum from falling over the dentition. In the sagittal plane, Inc. did not move to the front, but moved backward and downward. However, the tongue mainly moved to the front. Lt (masticatory side) moved to the front or backward, rather than downward. These movements suggest that the tongue picked up the gum on the dentition and held it in place on the dentition with the tongue acting like a wall. These coordinated movements between the tongue and mandible could indicate that the tongue placed the gum on the dentition.

Taniguchi et al. (2013) and Iida et al. (2023) measurements of the amount of tongue movement during mastication were greater than those measured in our study. Our studies used wire markers; therefore, the conductor may have limited the range of tongue movements. It is also possible that the VF images were distorted.

This research has several limitations. Because various devices were inserted in the oral cavity and the EMA markers (2 mm × 2 mm × 2 mm) have conductive wires (0.8 mm thickness), chewing behavior might have differed from normal chewing behavior. The EMA markers were attached to the tongue directly by using surgical adhesive, which might have affected the tongue movement because of mechanical and chemical stimulation. Additionally, the intraoral device (0.9 mm thickness) was inserted in the mandible to bundle the conductors. The conductors were passed through the right side, resulting in unilateral chewing on the left side only. However, we investigated whether these devices affect jaw movement (Supplemental experiment). In the results, the speed of jaw movement was affected by the application of the device, but the magnitude of jaw movement was not significantly affected. Therefore, we performed temporal analysis by normalizing based on the chewing cycle. In the future, we also need to verify how these devices affect tongue movements.

In this study, we analyzed the upper jaw as a reference. However, because the tongue is located in the mandible, it might be better to eliminate the influence of mandibular movement to analyze pure tongue movement. We will perform the analysis referred to mandibular point.

Furthermore, the results of this study were thought to be due to the movement of the tongue bodily movement and its deformation. The deformation, twist and rotated movement of tongue could be estimated by calculating changes of distance and angle based on the three-dimensional relationship between markers. Though we focused on the movement trajectory and distance of each part of the tongue in this study, we will analyze the deformation of the tongue in the future.

EMA cannot be used in conjunction with other devices that emit magnetic fields. Therefore, it was not possible to identify the position of the gum or the generation and strength of muscle activity. However, because the tongue pressure sensor sheet did not affect the EMA values, we measured tongue pressure at the same time. Although we did not analyze the huge amount of data gathered in this study, we plan to analyze the results of tongue pressure in the future and to examine further details of tongue movement during mastication.

When common solid foods are ingested, masticatory movement changes as it progresses (Hiiemae and Palmer, 1999). Food is first taken into the oral cavity and transported to the molar region by the tongue (Stage I Transport). The food is then crushed and mixed with saliva to form a bolus (Processing). The bolus formed between the dorsum of the tongue and the palate is transported little by little to the pharynx (Stage II Transport). In this study, we focused on tongue and jaw movement during pure chewing, so gum, which does not involve swallowing movements, was used as the test food. In future studies, the changes in tongue and jaw movements and their coordination from ingestion to swallowing should be investigated.

In this study, only young volunteers participated in this study. To date, tongue movement during mastication used EMA measurement in older people had not been reported. However, the tongue movement might be slow and the position of the tongue might be different from young people based on the results of EMA measurements during swallowing by Steele and Van Lieshout et al. (2009) and the results of tongue pressure measurements during swallowing by Tamine et al. (2010). We plan to conduct analyzes targeting older participants in the future.

Despite the limitations discussed, this study provides a detailed understanding of tongue movement during gum chewing. Clarifying the details of tongue movement during mastication should be useful in the evaluation and rehabilitation of tongue dysfunction, as well as in food development. The correlation of tongue pressure generation with tongue movement during mastication could be useful for diagnosing tongue movement disorders based on tongue pressure tests, resulting in more effective rehabilitation.

## 5 Conclusion

In this study, we measured tongue and jaw movement in three dimensions using EMA. The tongue moved downward and to the masticatory side following a similar pathway to the jaw. However, the jaw moved mainly backwards, whereas the tongue moved more forward than backward. Our results have clarified the sequence of tongue motion patterns during gum chewing.

## Data Availability

The raw data supporting the conclusions of this article will be made available by the authors, without undue reservation.
