# Supplementary figures and images for: Tongue and jaw movement assessed by 3D motion capture during gum chewing
Source: Front Physiol. 2024 Aug 28;15:1409005. doi: 10.3389/fphys.2024.1409005 (PMC11387162; doi:10.3389/fphys.2024.1409005)

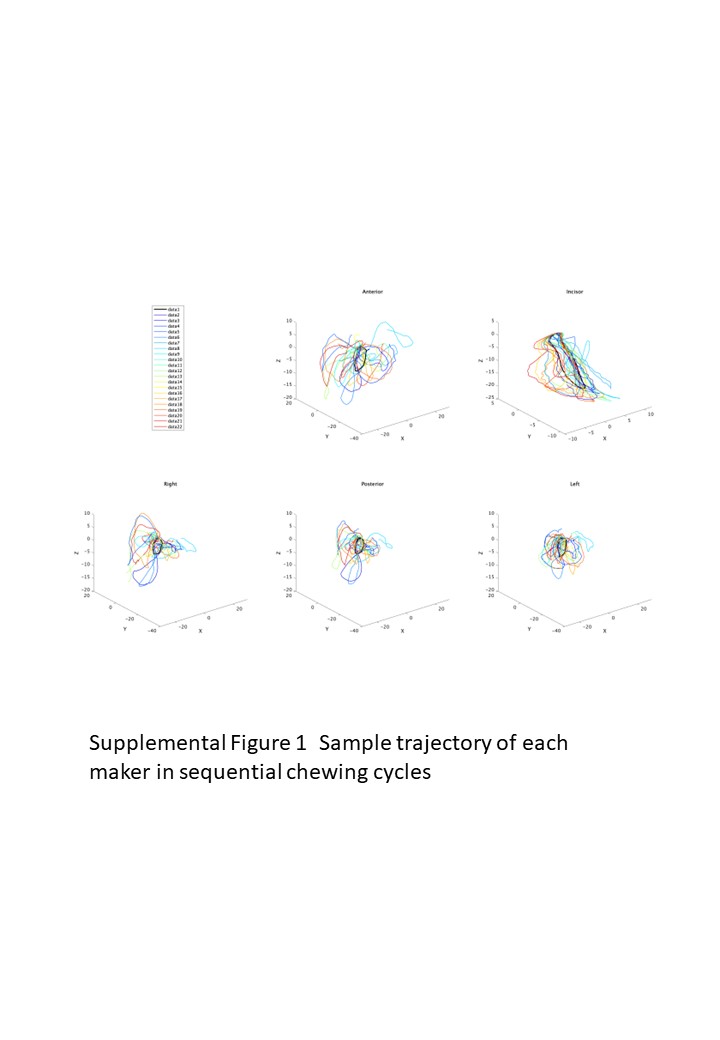

Supplement: Supplementary file 1 [file Image1.JPEG]

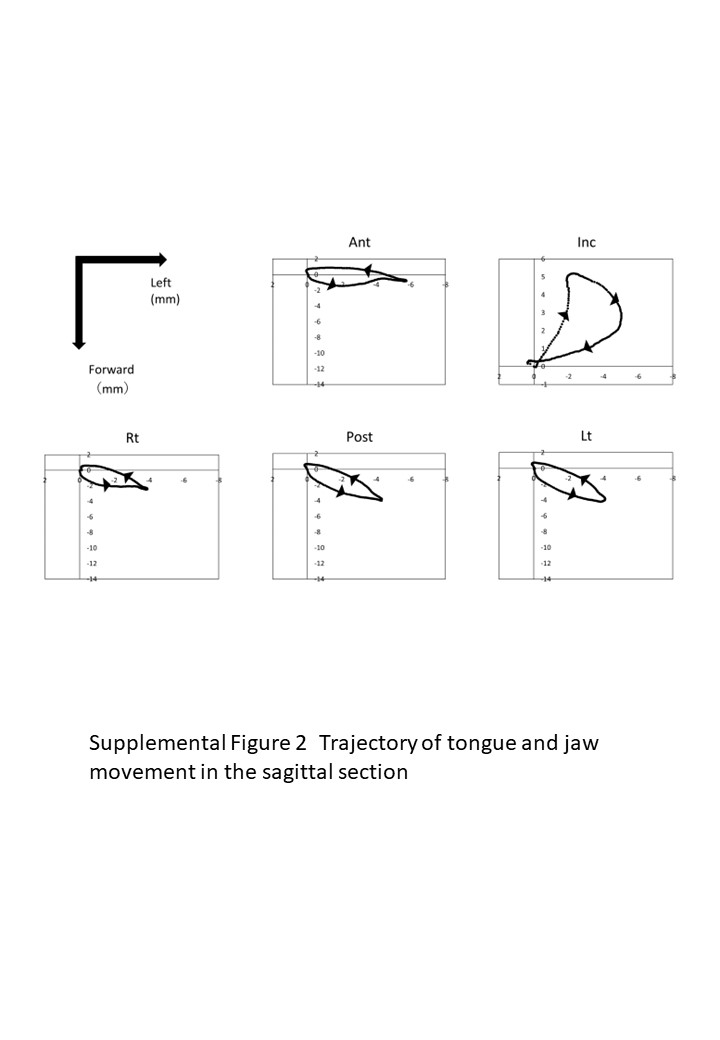

Supplement: Supplementary file 2 [file Image2.JPEG]
