# Supplementary material for: Tongue and jaw movement assessed by 3D motion capture during gum chewing
Source: Front Physiol. 2024 Aug 28;15:1409005. doi: 10.3389/fphys.2024.1409005 (PMC11387162; doi:10.3389/fphys.2024.1409005)
Supplement: Supplementary file 3 [file DataSheet1.PDF]

The difference of jaw movement during gum chewing with and without measuring device

<Objective>

This supplemental experiment performed to clarify the difference of jaw movement during gum chewing with and without measuring device.

<Material and methods>

The participants were three healthy volunteers (two men, one woman). The jaw movement was measured using mandibular kinesiograph (K7 evaluation system, Myotronics, WA, USA). The test food was instructed to chew a piece of tasteless and odorless gum (Saliva gum α, Tokyo Shizaisha Co. Ltd, Tokyo, Japan). The gum was softened in advance, and the participants instructed to hold the gum on the tongue and to chew 50 times only on left side after cue. The measurement was performed twice, i.e. with and without measuring devices. The measurement device was the same as that in the main experiment. That is, four EMA markers were attached to the tongue and an experimental intraoral plate was inserted to the mandible.

The chewing cycle duration and the amount of movement in the vertical, forward backward and lateral directions were calculated from the trajectory of jaw movement and compared with and without the measuring device using Student's t test. Furthermore, jaw movement was normalized by the chewing cycle, and the similarity of the trajectories w/wo devices was analyzed using ICC.

<Results>

The chewing cycle duration with devices were significantly longer than that without devices ($p=0.013$, Supplemental Experiment Figure 1). However, the vertical, forward backward and lateral movement did not show the significant differences ($p=0.141$, 0.743, 0.504, respectively, Supplemental Experiment Figure 1). The trajectories w/wo devices were very similar, and the high ICCs (2.1) were obtained (vertical: 0.950, forward backward: 0.715, lateral: 0.962, Supplemental Experiment Figure 2).

<Conclusion>

The speed of jaw movement was affected by the application of the device, but the magnitude of jaw movement was not significantly affected.

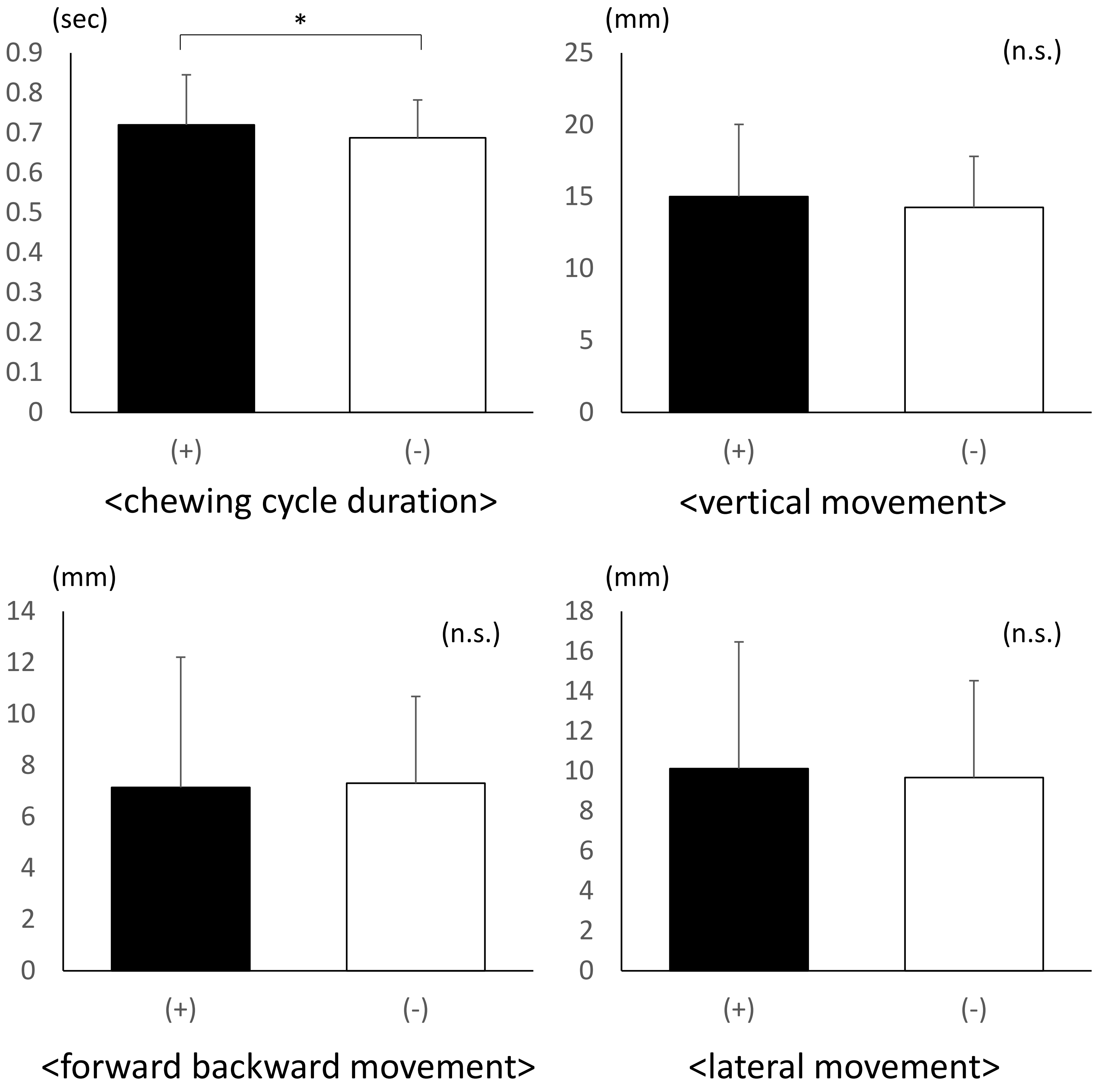

Supplemental Experiment Figure 1

The difference of jaw movement during gum chewing with and without measuring device

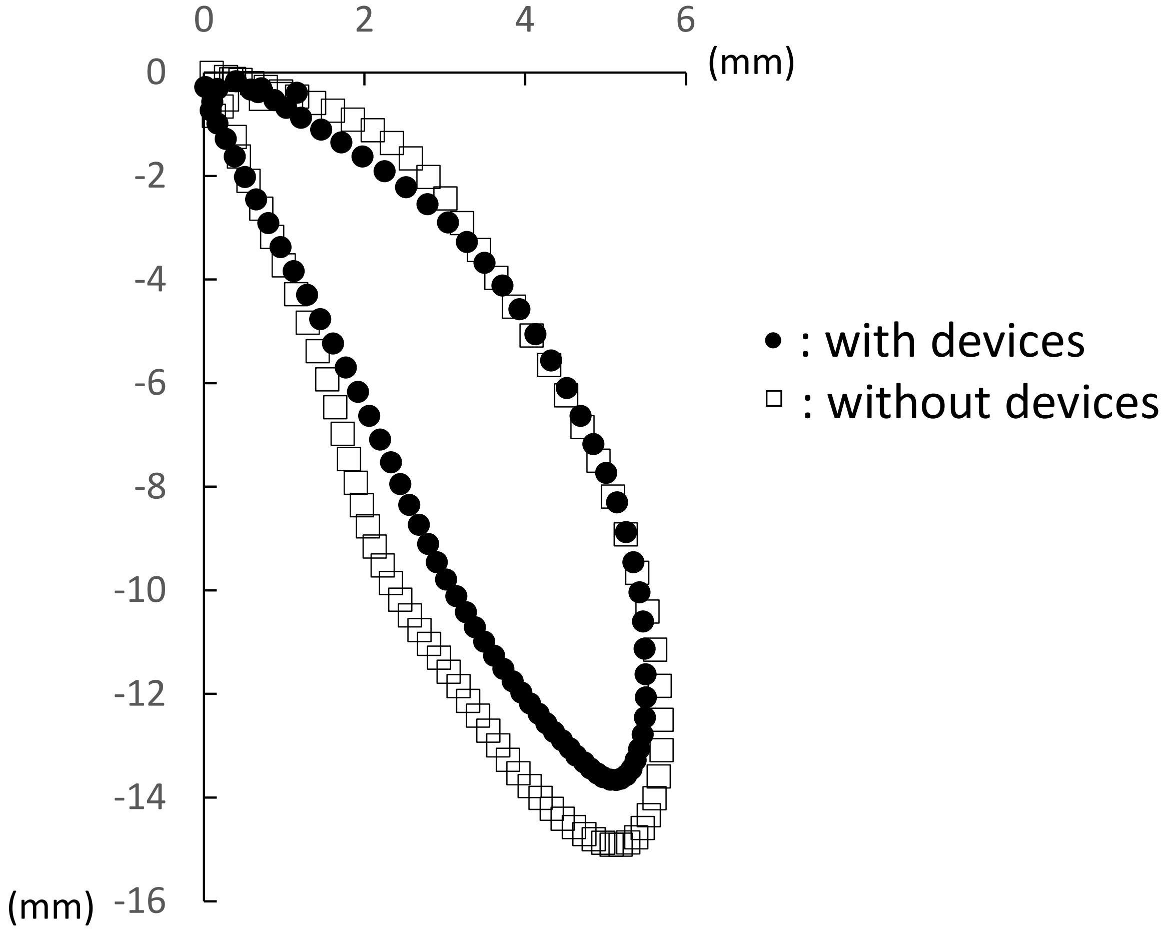

Supplemental Experiment Figure 2

The normalized jaw trajectories during gum chewing with and without measuring device
